# Knowledge and Predictors of Vitamin D Awareness Among Greek Women: A Cross-Sectional Study

**DOI:** 10.3390/diseases13020058

**Published:** 2025-02-15

**Authors:** Gavriela Voulgaridou, Fani Athanassiou, Eirini Kravvariti, Stephania Doulgeraki, Sousana K. Papadopoulou, Lambros E. Kokokiris

**Affiliations:** Department of Nutritional Sciences and Dietetics, School of Health Sciences, International Hellenic University, Alexander University Campus, 57400 Thessaloniki, Greece; gabivoulg@gmail.com (G.V.); faniathan@med.duth.gr (F.A.); stefdoulgeraki@ihu.gr (S.D.); sousana@ihu.gr (S.K.P.)

**Keywords:** vitamin D, knowledge, women, supplementation, attitudes

## Abstract

Background: Vitamin D plays a crucial role in bone health, calcium absorption, and immune function, yet significant misconceptions and knowledge gaps persist. This study aimed to assess knowledge regarding vitamin D among Greek women and identify factors associated with vitamin D knowledge. Methods: A cross-sectional study was conducted among 761 Greek women (mean age: 41.2 ± 7.7 years) using an online self-administered questionnaire designed in Google Forms. Participants were recruited via social media platforms to ensure a geographically diverse sample. Results: Overall, 57.4% of participants demonstrated knowledge of vitamin D’s functions, primarily linking it to bone health (34.4%) and immune function (26.8%). The multiple regression model identified significant predictors of knowledge of vitamin D’s functions, including age (OR = 1.041, 95% CI: 1.019–1.062, *p* < 0.001), weight (OR = 0.964, 95% CI: 0.938–0.992, *p* = 0.010), and frequent vitamin D testing (>2 times: OR = 2.280, 95% CI: 1.392–3.736, *p* = 0.001; once–twice: OR = 1.776, 95% CI: 1.111–2.829, *p* = 0.016). Furthermore, age (OR = 1.054, 95% CI: 1.027–1.081, *p* < 0.001), weight (OR = 0.987, 95% CI: 0.975–0.999, *p* = 0.028), higher city population (>50,000 citizens: OR = 1.598, 95% CI: 1.021–2.502, *p* = 0.040), frequent vitamin D testing (>2 times: OR = 2.616, 95% CI: 1.529–4.447, *p* < 0.003; one–two times: OR = 1.773, 95% CI: 1.052–2.989, *p* = 0.032), and children’s supplementation (OR = 1.414, 95% CI: 1.007–1.987, *p* = 0.046) were significant predictors of knowledge regarding diseases preventable by vitamin D. Conclusions: Greek women demonstrated moderate awareness of vitamin D’s functions, but significant knowledge gaps persist, particularly regarding its role in calcium absorption and dietary sources. Age, frequent vitamin D testing, and urban residence were significant predictors of knowledge. Targeted public health campaigns are essential to address misconceptions and improve vitamin D awareness and practices.

## 1. Introduction

Vitamin D, a fat-soluble steroid hormone, plays a crucial role in maintaining skeletal health by promoting the intestinal absorption of calcium and phosphorus [1]. Adequate vitamin D intake, particularly when combined with calcium, is well established to increase bone mineral density, reducing the risk of fractures and falls [2]. Severe vitamin D deficiency in children can lead to rickets, a metabolic bone disorder characterized by poor mineralization of developing bones [3]. In adults, low vitamin D levels manifest as osteomalacia and contribute to the development of osteoporosis [4]. Beyond its role in bone health, vitamin D also plays a critical part in immune function by enhancing innate immunity and regulating adaptive immune responses, fostering an anti-inflammatory environment that prevents harmful immune reactions [5]. Furthermore, it works synergistically with adequate protein intake to increase muscle mass by stimulating protein synthesis [6,7]. Vitamin D also influences cellular proliferation by modulating essential processes such as apoptosis, cell cycle regulation, and differentiation. These effects are mediated through cell-specific pathways that regulate key transcription factors and signaling mechanisms, vital for controlling programmed cell death, cell cycle checkpoints, and lineage-specific differentiation [8].

Despite its importance, vitamin D deficiency is highly prevalent worldwide. Severe deficiency, defined as levels below 30 mmol/L, affects approximately 15.7% of the global population, while deficiency (levels below 50 mmol/L) affects 47.9% [9]. Vitamin D deficiency and insufficiency are also common in pediatric populations globally, with rates ranging from 50 to 75%, even in developed countries [10,11]. Notably, females are 1.3 times more likely than males to have vitamin D concentrations below 30 mmol/L [9]. The prevalence of deficiency varies by region; in Greece, for example, rates range from 39.5% to 92.2%, depending on the cut-off values used [12]. Similarly, many Eastern Mediterranean countries, despite abundant sunshine, exhibit high rates of vitamin D deficiency [13].

This paradox is attributed to multiple contributing factors, including limited sun exposure, sunscreen use, darker skin pigmentation, insufficient dietary intake, socioeconomic status, and most importantly, a lack of knowledge about vitamin D and its dietary sources [14,15,16,17]. While many individuals may recognize that fatty fish and fortified foods are rich sources of vitamin D, studies indicate that knowledge does not always translate into behavior change, resulting in persistently low vitamin D intake in various populations [18,19].

Given the widespread prevalence and consequences of vitamin D deficiency, it is crucial to increase public awareness regarding its role in health and the risks associated with deficiency [20]. Several lines of evidence indicate an insufficient understanding of the importance of vitamin D, its dietary sources, and the need for supplementation [21,22,23]. However, addressing knowledge gaps alone may not be enough, as dietary behaviors are influenced by complex social and behavioral factors. This highlights the need for targeted interventions that extend beyond awareness campaigns, incorporating policy-driven strategies, supplementation programs, and behavior-focused nutrition initiatives to ensure effective prevention of deficiency.

Despite extensive global research on vitamin D deficiency, there is limited evidence focusing on the interplay between knowledge and actual health behaviors, particularly within the Greek population. The current study aims to assess the level of vitamin D knowledge among Greek women and identify the key factors influencing this knowledge. By addressing existing misconceptions and behavioral gaps, these findings are expected to help shape effective clinical and public health strategies to improve vitamin D levels and promote long-term health.

## 2. Materials and Methods

### 2.1. Study Design

A cross-sectional study design was used to collect data via an online questionnaire. The self-administered questionnaire was developed based on the existing literature [19,23] with some modifications incorporated. The questionnaire was provided in Greek. An expert panel reviewed the revised draft, and it was validated through a pilot study.

The study was approved by the ethical committee of the International Hellenic University (EC-IHU-No 85/2023). Participation in the survey was entirely voluntary, and consent was implied when respondents completed the online survey.

### 2.2. Study Population and Questionnaire

The study population consisted of women over 18 years old living in Greece. Responses from individuals under 18 were excluded. Google Forms were used to share questionnaires, which were distributed through social media platforms (i.e., Facebook, LinkedIn, and Twitter), mainly through personal outreach to friends, family members, acquaintances, and professional networks. Participants were also encouraged to share the survey within their own networks to facilitate a wider reach through snowball sampling. The survey was open to all and accessible via a direct web link, allowing voluntary participation without restrictions.

Data collection took place over a five-month period, from July 2024 to December 2024. Participants were provided with an information sheet before proceeding with the questionnaire. The sheet outlined the study’s objectives, confidentiality measures, and ethical considerations, including participants’ right to withdraw at any time, details on data storage duration, and information about the research investigators.

The questionnaire was developed based on the existing literature and reviewed by three experts in public health and nutrition to ensure content validity. A pilot study was conducted with 20 participants to assess the clarity, feasibility, and comprehensibility of the questions. Based on pilot study feedback, minor modifications were made to simplify certain items to enhance participant understanding. All pilot study responses were excluded from the final analysis, as their primary role was to refine the survey instrument. Reliability was evaluated using Cronbach’s alpha, which yielded a value of 0.70, indicating acceptable internal consistency.

The questionnaire consisted of 20 questions divided into four sections (Supplementary File S1). The first section included questions about the demographic and socioeconomic characteristics of the participants. The second section contained two open-ended, free-response questions to assess the women’s knowledge about vitamin D. These questions addressed the functions of vitamin D and its preventive or therapeutic role. If a woman provided at least one correct answer to each question, her response was considered correct, and she was categorized as having knowledge about vitamin D. Any other response, whether incorrect or absent, automatically categorized her as lacking knowledge. General responses, such as “yes”, “I know”, etc., were excluded from the analysis. The correct answers to the question regarding the functions of vitamin D were based on those outlined by the European Commission on nutrition and health claims [24]. For the question about its protective and/or therapeutic effects, only scientifically proven functions were accepted as correct. Functions for which research findings are still inconclusive were not considered correct answers [25]. Additionally, a third question assessed knowledge of vitamin D-rich foods. Women were asked to identify which food categories are good sources of vitamin D. They could select one or more categories from the following: fruits, vegetables, dairy products, eggs, seafood, meat, plant-based fats, cereal and bread, fish, liver, and chicken. The third section included questions regarding the frequency of consumption of foods containing vitamin D per week. Finally, the fourth section consisted of questions about self-supplementation and the supplementation of vitamin D in infants and children.

### 2.3. Sample Size

The sample size was determined using the formula (*n* = 10 k/p) specifically for logistic regression [26]. In this formula, k represents the number of covariates, and p denotes the proportion of instances in the population. A total of 12 covariates were included in the regression analysis. Based on prior studies suggesting a knowledge prevalence of approximately 0.5 (50%), a conservative approach was applied.

Initially, 801 women responded to the questionnaire, but 39 were excluded because their answers to the knowledge questions were too general, such as “yes”, “I know”, “it helps”, etc. Therefore, the final sample consisted of 761 women.

### 2.4. Statistical Analysis

The data collected were organized, categorized, and encoded. Statistical analysis was performed using the Statistical Package for Social Sciences (SPSS, version 27, Chicago, IL, USA). Descriptive analysis was conducted using frequencies and percentage distributions for categorical variables, while means and standard deviations were calculated for numeric variables. Descriptive characteristics of the study population were presented in tables. The chi-square test for independence was used to compare categorical variables between groups. Univariate associations between knowledge (dependent variable) and factors such as demographic and socioeconomic variables (independent variables) were assessed using binary logistic regression. Factors with a *p*-value < 0.2 were included in the final regression model. The results from the logistic regression were reported as odds ratios (ORs) with 95% confidence intervals (CIs). The significance level was set at α = 0.05.

## 3. Results

The mean age, weight, height, and BMI were 41.2 ± 7.7 years, 69.7 ± 14 kg, 1.7 ± 0.06 m, and 25.1 ± 4.6, respectively (Table 1). Additionally, 72.8% of the participants had a higher educational level, and 50.7% were residents of a city with a population of over 50,000 inhabitants.

A slight majority of the women had knowledge about the functions of vitamin D (57.4%, Table 2). Of these, 34.4% knew that vitamin D helps maintain bone strength and hardness, 26.8% knew it supports normal immune function, and 10% were aware that it stimulates calcium and phosphorus absorption. Incorrect responses regarding vitamin D’s role included helping with depression and/or improving mood (7.1%), supporting brain function (2.6%), benefiting the nervous system (2.5%), reducing the risk of cardiovascular diseases (CVDs) (2.2%), and assisting with blood sugar control (1.8%).

In contrast, as shown in Table 3, most women (66%) were unaware of the diseases that vitamin D can prevent and/or treat. Osteoporosis was the most known disease (31.5%), while a smaller proportion (5.5%) were aware of rickets. Some women mistakenly believed that vitamin D could prevent or treat autoimmune diseases (10.4%), CVDs (7.8%), depression and other psychiatric disorders (6.3%), and cancer (4.6%).

Women were asked to choose from 11 food categories which ones they believed were good sources of vitamin D (Table 4). Approximately, half of the sample selected both eggs (47.7%) and fish (48.6%) as vitamin D sources. Additionally, 38.8% believed dairy products, 25.2% believed vegetables, and 22.1% believed liver to be good sources of vitamin D. Notably, 24.4% of the women incorrectly believed that fruit is a source of vitamin D. Significant associations were found between beliefs and knowledge levels for several food categories (*p* < 0.001, Table 4). Specifically, participants with knowledge about vitamin D’s functions were more likely to identify eggs (*p* < 0.001), dairy products (*p* < 0.001), fish (*p* < 0.001), and liver (*p* < 0.001) as good sources of vitamin D. Both meat (*p* < 0.001) and plant-based fats (*p* = 0.006) were more commonly identified as sources by women with vitamin D knowledge, even though these are not reliable sources of vitamin D. Conversely, those without knowledge were more likely to mistakenly identify fruits (*p* = 0.002) and cereals/bread (*p* < 0.001) as sources of vitamin D (Table 4).

Most of the women (49.5%) were tested for vitamin D more than twice in their lives, and 55.7% had deficient or insufficient levels of vitamin D (Table 5). Additionally, 55.5% of participants supplemented during their lives, and 33.9% supplemented specifically during pregnancy. Of the women, 33.5% supplemented their infants, and 40.3% supplemented their children.

A univariate and multivariate logistic regression analysis was conducted to identify predictors of knowledge about the role of vitamin D among the participants (Table 6). The univariate analysis indicated that age was positively associated with vitamin D knowledge (OR = 1.037, 95% CI = 1.018–1.057, *p* < 0.001), suggesting that older participants were more likely to possess knowledge about vitamin D. Additionally, weight showed a slight inverse association (OR = 0.986, 95% CI = 0.976–0.997, *p* = 0.009), implying that lower weight was marginally associated with increased knowledge. Monthly income was a significant predictor; those with an income between EUR 2000 and EUR 3000 had increased knowledge (OR = 1.931, 95% CI = 1.231–3.028, *p* = 0.004) compared to those with an income < EUR 1000, while those earning over EUR 3000 also showed a significant positive association (OR = 1.715, 95% CI = 1.032–2.850, *p* = 0.037). Participants who were tested once or twice for vitamin D levels were 1.7 times more knowledgeable than those who were never tested (OR = 1.720, 95% CI = 1.146–2.581, *p* = 0.009), while those tested more than twice had over twice the likelihood of knowledge compared to those never tested (OR = 2.292, 95% CI = 1.587–3.310, *p* < 0.001). A diagnosis of vitamin D deficiency also significantly increased the likelihood of knowledge (OR = 1.433, 95% CI = 1.073–1.915, *p* = 0.015). Vitamin D supplementation outside of pregnancy was positively associated with knowledge (OR = 1.751, 95% CI = 1.309–2.342, *p* < 0.001), and participants who took a supplement during pregnancy also showed a marginally significant increase in knowledge (OR = 1.365, 95% CI = 1.004–1.856, *p* = 0.047). Additionally, participants who gave supplements to their children without a medical indication were 1.5 times more likely to have vitamin D knowledge (OR = 1.457, 95% CI = 1.084–1.959, *p* = 0.013).

The multivariate analysis found significant predictors of knowledge, including age (OR = 1.041, 95% CI = 1.019–1.062, *p* < 0.001), testing frequency (tested once or twice, OR = 1.776, 95% CI = 1.111–2.839, *p* = 0.016; tested more than twice, OR = 2.280, 95% CI = 1.392–3.736, *p* = 0.001), and an income of EUR 2000–3000 (OR = 1.530, 95% CI = 0.937–2.498, *p* = 0.089).

The univariate and multivariate logistic regression analyses in Table 7 explored predictors of knowledge about diseases associated with vitamin D deficiency. In the univariate analysis, age showed a strong positive association with knowledge of these diseases (OR = 1.061, 95% CI = 1.039–1.083, *p* < 0.001), indicating that older participants were more likely to be knowledgeable. Weight demonstrated a slight inverse association (OR = 0.992, 95% CI = 0.981–1.003, *p* = 0.143), though this was not statistically significant. Participants from larger cities (population > 50,000) exhibited a significantly higher likelihood of knowledge compared to those from smaller cities (OR = 1.956, 95% CI = 1.291–2.962, *p* = 0.002), and this association remained significant in the multivariate model (OR = 1.598, 95% CI = 1.021–2.502, *p* = 0.040). Additionally, participants with higher education levels were more knowledgeable (OR = 1.463, 95% CI = 1.032–2.074, *p* = 0.033). Monthly income was also a significant predictor in the univariate analysis. Participants earning between EUR 2000 and EUR 3000 demonstrated greater knowledge (OR = 1.788, 95% CI = 1.105–2.892, *p* = 0.018), while those earning over EUR 3000 showed an even stronger association (OR = 2.187, 95% CI = 1.283–3.727, *p* = 0.004). Testing frequency was a particularly strong predictor of knowledge. Participants tested once or twice were 1.6 times more likely to have knowledge than those never tested (OR = 1.610, 95% CI = 1.018–2.548, *p* = 0.042), and those tested more than twice were over 2 times as likely to be knowledgeable (OR = 2.304, 95% CI = 1.526–3.478, *p* < 0.001). A diagnosis of vitamin D deficiency was also associated with increased knowledge (OR = 1.422, 95% CI = 1.048–1.930, *p* = 0.024). Vitamin D supplementation outside of pregnancy showed a significant association with knowledge (OR = 1.480, 95% CI = 1.090–2.010, *p* = 0.012). Additionally, participants who gave supplements to their children without a medical indication demonstrated greater knowledge (OR = 1.420, 95% CI = 1.048–1.924, *p* = 0.024).

The multivariate analysis showed that age was a significant predictor, with each additional year associated with a 5.4% increase in knowledge (OR = 1.054, 95% CI = 1.027–1.081, *p* < 0.001). Weight, in the multivariate model, showed a marginal association (OR = 0.987, 95% CI = 0.975–0.999, *p* = 0.028). City population was also a significant predictor, with participants from larger cities (>50,000 population) showing greater knowledge (OR = 1.598, 95% CI = 1.021–2.502, *p* = 0.040). Participants who earned over EUR 3000 showed a marginally significant association with knowledge (OR = 1.637, 95% CI = 0.923–2.902, *p* = 0.092), whereas those who earned EUR 2000–3000 did not differ significantly from the reference group in the multivariate model (*p* = 0.516). Participants tested once or twice had higher knowledge levels (OR = 1.773, 95% CI = 1.052–2.989, *p* = 0.032), while those tested more than twice were 2.6 times more likely to have knowledge about vitamin D-related diseases (OR = 2.616, 95% CI = 1.529–4.447, *p* < 0.001). Participants who gave supplements to their children without a medical need continued to show a significant association with knowledge (OR = 1.414, 95% CI = 1.007–1.987, *p* = 0.046).

## 4. Discussion

**Figure 1:**
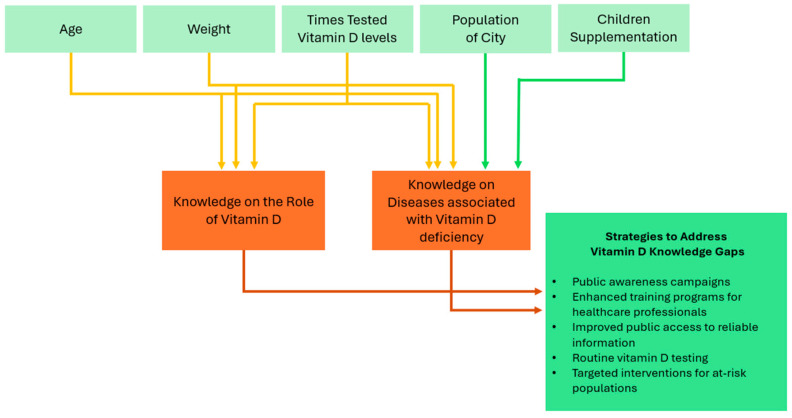
Overview of predictors of vitamin D knowledge and public health strategies.

These results suggest that knowledge about vitamin D is significantly associated with accurate beliefs about dietary sources of this nutrient. Our results suggest that knowledge was positively associated with age, higher income, frequent vitamin D testing, and supplementation practices (Figure 1).

Our study revealed that 57.4% of participants demonstrated knowledge of vitamin D’s functions. This aligns with findings from several studies in the United Arab Emirates [27], Saudi Arabia [28], and the UK [23,29,30]. In a study conducted in the United Kingdom, 54% of participants exhibited low knowledge about the basic symptoms of vitamin D deficiency [29], whereas another UK-based study reported a mean knowledge score of 56.6% [19], and a more recent study found a higher knowledge level of 75% [30]. In contrast, compared to other populations, our results demonstrated a higher mean knowledge score than those observed in Canada (27%) [31] and China (32.2%) [32].

Our findings demonstrated a relatively higher awareness of dietary sources of vitamin D, with 47.7% and 48.6% of participants correctly identifying eggs and fish, respectively. However, misconceptions were also evident, as 24.4% of participants mistakenly cited fruits as a source of vitamin D. This aligns with findings from other studies that highlight persistent gaps in knowledge regarding dietary sources of vitamin D [33,34,35]. For instance, Ho-Pham and Nguyen (2012) reported low awareness among urban populations in Vietnam, where participants exhibited confusion about food sources of vitamin D [33]. Similarly, an Australian study found that only 33% of participants correctly identified dietary sources of this vitamin [34]. In the UK, oily fish—considered the best dietary source—was correctly identified by only half of the participants, reflecting a similar level of awareness [19]. Comparatively, Alfadly et al. (2024) reported significantly lower awareness, with only 30.95% of participants identifying eggs, 14.88% recognizing fatty fish, and 26.78% correctly identifying dairy milk as vitamin D sources [36]. Similarly, Aljefree et al. (2017) highlighted considerable gaps in knowledge, where only a small proportion of participants recognized milk, cheese, or fortified foods as sources of vitamin D, while some incorrectly believed that fruits and vegetables could prevent deficiency [37]. These misconceptions may stem from the widespread dissemination of unverified information through media and the lack of adequate public education from scientifically validated sources. To address these misunderstandings, targeted educational campaigns are essential to inform the public about the evidence-based roles of vitamin D, while clarifying the distinction between hypotheses and scientifically proven data.

In a related study, Alibrahim et al. (2024) in Syria reported that 84.6% of participants recognized vitamin D’s role in maintaining calcium and phosphate levels, yet 62% disagreed with the statement that vitamin D is found only in animal meat, suggesting ongoing confusion about plant-based sources [21]. Similarly, Uzrail et al. (2021) in Jordan revealed that 20.9% and 16.8% of respondents incorrectly cited fruits and vegetables, respectively, as sources of vitamin D [38]. While the Jordanian study showed slightly better awareness of food sources such as milk and fish, misconceptions about plant-based sources persisted across populations. These gaps underscore the importance of targeted public health campaigns to correct misunderstandings and promote accurate knowledge of vitamin D sources across diverse populations.

Our study revealed that 57.4% of participants demonstrated knowledge of vitamin D’s functions, primarily linking it to bone health (34.4%) and immune function (26.8%). However, 66% were unaware of diseases that could be prevented or treated with adequate vitamin D levels, with osteoporosis being the most recognized condition (31.5%). A significant proportion of participants (42.6%) lacked sufficient knowledge about the broader importance of vitamin D, and misconceptions were common, such as associating vitamin D with cardiovascular disease (7.8%) and depression (6.3%). Similarly, a qualitative study conducted in Saudi Arabia reported that participants had reasonable knowledge of vitamin D’s role in bone health but demonstrated limited awareness of deficiency risks, particularly osteoporosis and rickets [37].

Comparable findings were observed in India and Vietnam, where 88% [39] and 88.5% [33] of participants, respectively, demonstrated good awareness of vitamin D’s benefits for bone health but lacked understanding of its preventive role in specific conditions, such as osteoporosis and rickets. A UK study also highlighted that 82% of participants were aware of vitamin D’s role in bone health; however, fewer participants associated it with the prevention of osteoporosis and rickets [19]. Similarly, Vu et al. (2010) found that 18% of participants in Australia failed to recognize any confirmed benefits of vitamin D for bone health [34]. These findings underscore a common pattern across populations: while general awareness of vitamin D’s connection to bone health is relatively high, knowledge gaps persist regarding the specific risks of deficiency and its broader preventive role. Addressing these gaps through targeted educational campaigns could improve public understanding and health outcomes globally.

We also revealed that only 10% of participants correctly identified vitamin D’s role in calcium and phosphorus absorption, highlighting significant knowledge gaps regarding this critical function. In contrast, the Syrian study reported much higher awareness, with 91.4% and 84.6% of participants correctly recognizing vitamin D’s importance in maintaining calcium and phosphate balance, respectively [21]. Similarly, Malek et al. (2023) demonstrated that 75.8% of participants acknowledged vitamin D’s role in regulating calcium and phosphate levels and preventing bone diseases such as rickets [40]. These disparities underscore considerable variation in public knowledge across different populations. While both Syrian studies indicate a strong baseline understanding of vitamin D’s role in mineral homeostasis, our findings emphasize the urgent need for targeted educational initiatives to improve foundational knowledge and address misconceptions within our population.

Misconceptions about the effects of vitamin D were common in our study, with 7.8% of participants incorrectly associating it with cardiovascular diseases (CVDs) and 6.3% linking it to mood disorders such as depression. Similar findings were reported by Alfady et al. (2024) and Babelghaith et al. (2017), where 45.5% and 48.4% of participants, respectively, believed that vitamin D deficiency could lead to conditions such as CVDs and cancer [36,41]. Although some studies suggest potential associations between insufficient vitamin D levels and these conditions, these claims remain unsupported by official guidelines or conclusive evidence. Such findings highlight significant gaps in public knowledge and the prevalence of misinformation regarding vitamin D’s broader health effects beyond its well-established roles in bone health, calcium absorption, and immune function. Addressing these misconceptions is essential to ensure that public health messaging focuses on scientifically validated benefits, reducing confusion, and promoting an accurate understanding of vitamin D’s importance.

We found that 55.7% of participants had been diagnosed with vitamin D deficiency, and 55.5% reported taking vitamin D supplements. Additionally, 33.5% of women supplemented their infants, while 40.3% provided supplements to their children. In contrast, Zadka et al. (2018) reported that vitamin D was not supplemented among over 60% of children and more than 70% of their mothers, reflecting significant gaps in supplementation practices [23]. Similarly, Alibrahim et al. (2024) in Syria found limited use of vitamin D supplements, with supplementation practices largely influenced by socioeconomic factors [21]. In Saudi Arabia, Kambal et al. (2023) also highlighted suboptimal supplementation rates, with only 32.4% of female participants reporting vitamin D intake [28]. These findings underscore the variability in supplementation practices across different regions and populations, influenced by socioeconomic conditions, cultural norms, and access to healthcare. Targeted interventions that address these barriers and promote vitamin D supplementation—particularly among mothers and young children—are crucial for improving health outcomes and preventing deficiency-related conditions.

Age was significantly associated with knowledge about the benefits of vitamin D. This finding aligns with the results of other studies, such as those of Alfadly et al. (2024), which also identified a significant association between age and knowledge of vitamin D benefits [36]. Conversely, other studies did not find age to be associated with vitamin D knowledge [28,34,42]. Notably, Alamoudi et al. (2019) reported no significant association between knowledge sources or overall knowledge (benefits and sources combined) and age [42]. In addition, our study revealed significant associations between age and knowledge of diseases related to vitamin D deficiency, further supporting the role of age as an influential factor in vitamin D awareness. Similar findings were reported in the UAE study, where Emirati participants demonstrated significantly higher knowledge of vitamin D compared to tourists, with nationality and age emerging as significant predictors of knowledge [27].

Weight emerged as a significant predictor of knowledge regarding both vitamin D’s functions and diseases preventable by vitamin D. Specifically, lower weight was associated with better knowledge levels. Although there is limited literature directly linking body weight to vitamin D knowledge, it is well documented that individuals with lower weight or those at risk of conditions such as osteoporosis are more likely to receive medical advice or health education regarding vitamin D’s role in bone health and calcium metabolism [43]. For example, vitamin D deficiency is frequently observed in populations with low body weight, particularly among postmenopausal women [44], who are targeted for bone health interventions and nutritional education. Additionally, weight-conscious individuals may engage in more frequent health monitoring and nutritional counseling, which could contribute to healthier dietary habits.

Based on our results, educational attainment was not a significant determinant of vitamin D knowledge. In contrast, several other studies demonstrated a clear association between higher education levels and greater awareness of vitamin D [19,42,45]. For example, Al-Agha et al. (2016) in Jeddah reported that highly educated parents outperformed those with lower education levels in correctly answering questions about vitamin D [45]. Similarly, Alibrahim et al. (2024) in Syria found that participants with Ph.D. degrees had significantly higher knowledge scores compared to those with lower educational levels [21]. Alamoudi et al. (2019) in Saudi Arabia also observed that individuals with higher education demonstrated better awareness of vitamin D’s functions, highlighting a knowledge gap among less educated groups [42]. These contrasting findings, alongside ours, suggest that while education can be a strong predictor of vitamin D knowledge in certain contexts, other factors such as age, socioeconomic status, or access to health information may play a more significant role in shaping awareness in specific populations. This highlights the need for tailored public health initiatives that address knowledge gaps across diverse educational backgrounds.

We found that residence, particularly in cities with populations exceeding >50,000, was significantly associated with higher knowledge of diseases related to vitamin D deficiency, with participants being 1.6 times more likely to demonstrate such knowledge compared to those living in smaller cities. This aligns with findings from Al-Ghraibawi et al. (2019) and Kambal et al. (2023), which reported that individuals in urban areas exhibit greater awareness and knowledge about vitamin D compared to rural populations [28,46]. These differences are likely attributable to better access to educational resources, healthcare services, and public health initiatives in urban settings. These findings underscore the need for targeted awareness campaigns in rural and less populated areas to address disparities in vitamin D knowledge and promote equitable health education.

Additionally, we identified that having been tested for vitamin D levels was an independent predictor of vitamin D knowledge, with tested participants demonstrating significantly greater awareness of its role and health benefits. Similarly, a study among university students in Pakistan found that those who had undergone vitamin D testing were more likely to recognize bone-related health benefits and factors affecting vitamin D levels [47]. Supporting this, a UK study reported that 51% of respondents had undergone vitamin D testing, with many advocating for its inclusion in routine health checks to raise awareness and improve public knowledge about deficiency and its prevention [30]. These findings collectively emphasize the dual role of vitamin D testing, not only as a diagnostic tool but also as a valuable opportunity to educate individuals about the health benefits of vitamin D and strategies to prevent its deficiencies.

This study offers several notable strengths. To the best of our knowledge, this is the first study conducted in Greece to comprehensively evaluate knowledge, practices, and misconceptions regarding vitamin D among Greek women. The study also benefits from a large sample size, ensuring robust statistical power to analyze multiple dimensions of vitamin D knowledge, including its benefits, dietary sources, and supplementation practices. However, the study has some limitations. First, the cross-sectional design precludes any causal inferences about the relationships between predictors, such as age or prior testing, and vitamin D knowledge. Second, the reliance on self-reported data introduces the potential for recall bias and social desirability bias, where participants may have over- or under-reported their knowledge or practices. Furthermore, certain unmeasured confounders, such as access to healthcare, exposure to educational campaigns, and sun exposure habits, were not fully assessed but may influence knowledge levels. Another limitation of this study is the use of social media platforms for questionnaire distribution. While this approach allowed for a wide and rapid reach, it inherently introduced a selection bias by excluding individuals who do not use or have limited access to social media, such as older adults. Lastly, while the study evaluated awareness of dietary sources of vitamin D, it did not assess actual dietary intake or vitamin D status, such as serum 25(OH)D levels. Measuring these would provide a more complete understanding of vitamin D deficiency and related practices.

## 5. Strategies for Improving Vitamin D Awareness

To enhance public understanding of vitamin D and correct misconceptions, targeted educational initiatives should be prioritized. National health agencies could implement awareness campaigns utilizing social media, television, and community programs to ensure that accurate information reaches a broad audience. Schools should integrate vitamin D education into health and nutrition curricula to promote awareness from an early age.

Healthcare professionals play a critical role in bridging the knowledge gap. Enhancing training programs and continuing medical education on vitamin D can equip physicians, dietitians, and pharmacists with the necessary knowledge to provide accurate recommendations to patients. Encouraging routine vitamin D assessments, especially among vulnerable populations such as pregnant women and older adults, can further help prevent deficiencies. These combined efforts will contribute to a more informed population and improved public health outcomes related to vitamin D.

However, it is important to note that these findings specifically apply to Greek women, as the study population was limited to this demographic. Cultural, dietary, and healthcare system factors unique to Greece may influence the generalizability of these results to other populations. Future research should explore whether similar knowledge gaps exist in different populations to determine if these findings are consistent across diverse sociocultural contexts.

## 6. Conclusions

This study highlights moderate levels of knowledge regarding vitamin D’s roles, with participants primarily linking it to bone health and immune function. However, significant misconceptions persist, particularly regarding dietary sources, such as fruits, and their role in calcium and phosphorus absorption. Gaps in awareness of diseases preventable by adequate vitamin D levels, such as osteoporosis and rickets, were also evident.

Age, frequent vitamin D testing, supplementation practices, and urban residence were identified as key predictors of knowledge, while educational attainment did not significantly influence awareness. Addressing these knowledge gaps requires a multifaceted approach that includes improved public access to reliable information, routine testing, and greater engagement with healthcare providers. Future research should explore the impact of educational interventions and monitor vitamin D practices to better support populations at risk of deficiency.

## Figures and Tables

**Table 1:** Characteristics of Greek women (*n* = 761) regarding morphometric and demographic variables (mean ± SD) and frequency values (*n*, %).

Variables	
Morphometric	Mean (±sd)
Age (y)	41.2 ± 7.7
Weight (kg)	69.7 ± 14
Height (m)	1.7 ± 0.06
BMI (kg/m^2^)	25.1 ± 4.6
Demographic/Socioeconomic	*n* (%)
Population of the city of residence (%)	
100–20,000	159 (20.9)
20,000–50,000	216 (28.4)
>50,000	386 (50.7)
Educational level (%)	
Primary and Secondary education	207 (27.2)
Higher education	554 (72.8)
Number of children < 18 years old (%)	
0 children	33 (4.3)
1 child	334 (43.9)
2 children	332 (43.6)
>3 children	62 (8.1)
Occupation (%)	
Civil servant	186 (24.4)
Private servant	321 (42.2)
Household	146 (19.2)
Medical–Pharmaceutical profession	39 (5.1)
Other	69 (9.1)
Monthly salary (EUR) (%)	
<1000	158 (20.8)
1000–2000	343 (45.1)
2000–3000	159 (20.9)
>3000	101 (13.3)

**Table 2:** Assessment of the level of knowledge about the function of vitamin D among Greek women.

Vitamin D’s Functions	Frequency *n* (%)
Women	
With knowledge	437 (57.4)
Without knowledge	324 (42.6)
Type of correct responses	
Calcium and phosphorus absorption	76 (10)
Regulates normal blood calcium levels	5 (0.7)
Normal bones maintenance	262 (34.4)
Maintaining normal muscle function	16 (2.1)
Healthy teeth maintenance	18 (2.4)
Maintenance of normal immune function	204 (26.8)
Maintaining normal immune function in children	1 (0.1)
Normal bone development in children	6 (0.8)
Type of wrong responses	
Prevents CVDs	17 (2.2)
Helps with depression/Improves mood	54 (7.1)
Give energy	16 (2.1)
Brain function	20 (2.6)
Weight maintenance	5 (0.7)
Blood sugar control	14 (1.8)
Anti-cancer action	5 (0.7)
Nervous system	19 (2.5)

CVDs = cardiovascular diseases.

**Table 3:** Assessment of the level of knowledge about the diseases prevented or treated with vitamin D among Greek women.

Knowledge of Diseases Prevented or Treated by Vitamin D	Frequency *n* (%)
Women	
With knowledge	259 (34)
Without knowledge	502 (66)
Type of correct responses	
Rickets	42 (5.5)
Hypoparathyroidism	3 (0.4)
Osteoporosis	240 (31.5)
Type of wrong responses	
Thyroid diseases	18 (2.4)
Depression and other psychiatric diseases	48 (6.3)
Muscle-related diseases	13 (1.7)
Autoimmune diseases	79 (10.4)
CVDs	59 (7.8)
Cancer	35 (4.6)
Neurological diseases	14 (1.8)

CVDs = cardiovascular diseases.

**Table 4:** Comparison of Greek women’s knowledge of good sources of vitamin D according to their different levels of knowledge about vitamin D’s functions.

Food Categories	Total Responses of Women	Responses of Women with Knowledge on Vit. D’s Functions	Responses of Women Without Knowledge on Vit. D’s Functions	*p*-Value
Fruits	186 (24.4)	89 (20.4)	97 (29.9)	0.002
Dairies	295 (38.8)	232 (53.1)	63 (19.4)	<0.001
Eggs	363 (47.7)	278 (63.6)	85 (26.2)	<0.001
Plant-based fat	111 (14.6)	77 (17.6)	34 (10.5)	0.006
Cereals/bread	182 (23.9)	129 (29.5)	53 (16.4)	<0.001
Fish	370 (48.6)	276 (63.2)	94 (29.0)	<0.001
Liver	168 (22.1)	130 (29.7)	38 (11.7)	<0.001
Meat	98 (12.9)	78 (17.8)	20 (6.2)	<0.001
Seafood	96 (12.6)	64 (14.6)	32 (9.9)	0.050
Vegetables	192 (25.2)	104 (23.8)	88 (27.2)	0.291
Chicken	24 (3.2)	18 (4.1)	6 (1.90)	0.077
Do not know	164 (21.6)	43 (9.8)	121 (37.3)	<0.001

*p* value: χ^2^ test of independence, α = 0.05.

**Table 5:** Assessment of the behavior about vitamin D testing, deficiency, and supplementation practices among Greek women.

Questions	Frequency *n* (%)
How many times have you been tested for vitamin D levels in your body?	
None	172 (22.6)
Once–Twice	212 (27.8)
>2 times	377 (49.5)
Have you been diagnosed with vitamin D deficiency or insufficiency in your body at least once in your lifetime?	
No	337 (44.3)
Yes	424 (55.7)
Have you been taking a vitamin D supplement for at least one period outside of pregnancy?	
No	339 (44.5)
Yes	422 (55.5)
Did you take a supplement for vitamin D during pregnancy?	
No	206 (66.1)
Yes	258 (33.9)
Have you ever given vitamin D supplements to your infant?	
No	506 (66.5)
Yes	255 (33.5)
Have you ever given vitamin D supplements to your children without need?	
No	454 (59.7)
Yes	307 (40.3)
In which age period of children is there the greatest need for vitamin D?	
0–6 years old	272 (35.7)
7–12 years old	76 (10)
13–18 years old	86 (11.3)
I do not know	327 (43)

**Table 6:** The effect of selected predictors on knowledge about the role of vitamin D among Greek women.

		Univariate Analysis			Multivariate Analysis	
	OR	95% CI	*p*-Value	OR	95% CI	*p*-Value
Morphometric						
Age (y)	1.037	1.018–1.057	<0.001	1.041	1.019–1.062	<0.001
Weight (kg)	0.986	0.976–0.997	0.009	0.964	0.938–0.992	0.010
BMI (kg/m^2^)	0.972	0.942–1.002	0.071	1.067	0.981–1.160	0.130
City population						
100–20,000	Ref					
20,000–50,000	1.055	0.700–1.591	0.798	0.815	0.525–1.264	0.361
>50,000	1.405	0.968–2.039	0.074	1.102	0.736–1.651	0.637
Educational level						
Primary and Secondary	Ref					
High level	1.377	0.999–1.898	0.051	1.089	0.762–1.557	0.638
Monthly salary (EUR)						
<1000	Ref					
1000–2000	1.456	0.997–2.125	0.052	1.336	0.892–2.000	0.160
2000–3000	1.931	1.231–3.028	0.004	1.530	0.937–2.498	0.089
>3000	1.715	1.032–2.850	0.037	1.480	0.587–2.557	0.159
Times tested for vitamin D levels						
None	Ref					
Once–Twice	1.720	1.146–2.581	0.009	1.776	1.111–2.839	0.016
>2	2.292	1.587–3.310	<0.001	2.280	1.392–3.736	0.001
Diagnosed with vitamin D deficiency						
No	Ref					
Yes	1.433	1.073–1.915	0.015	0.693	0.442–1.085	0.109
Vitamin D supplementation						
No	Ref					
Yes	1.751	1.309–2.342	<0.001	1.434	0.966–2.129	0.074
Vitamin D supplementation during pregnancy						
No	Ref					
Yes	1.365	1.004–1.856	0.047	1.217	0.866–1.710	0.257
Supplementing children without needed of vitamin D						
No	Ref					
Yes	1.457	1.084–1.959	0.013	1.285	0.935–1.764	0.122

**Table 7:** The effect of selected predictors on knowledge about diseases associated with vitamin D deficiency among Greek women.

		Univariate Analysis			Multivariate Analysis	
	OR	95% CI	*p*-Value	OR	95% CI	*p*-Value
Morphometric						
Age (yrs)	1.061	1.039–1.083	<0.001	1.054	1.027–1.081	<0.001
Weight (kg)	0.992	0.981–1.003	0.143	0.987	0.975–0.999	0.028
BMI (kg/m^2^)	0.986	0.954–1.019	0.400			
City population						
100–20,000	Ref					
20,000–50,000	1.475	0.931–2.337	0.098	1.160	0.713–1.888	0.550
>50,000	1.956	1.291–2.962	0.002	1.598	1.021–2.502	0.040
Educational level						
Primary and Secondary	Ref					
High level	1.463	1.032–2.074	0.033	1.253	0.851–1.845	0.253
Monthly salary						
<1000	Ref					
1000–2000	1.507	0.988–2.300	0.057	1.280	0.818–2.005	0.280
2000–3000	1.788	1.105–2.892	0.018	1.189	0.705–2.008	0.516
>3000	2.187	1.283–3.727	0.004	1.637	0.923–2.902	0.092
Times tested for vitamin D levels						
None	Ref					
Once–Twice	1.610	1.018–2.548	0.042	1.773	1.052–2.989	0.032
>2	2.304	1.526–3.478	<0.001	2.616	1.529–4.447	<0.001
Diagnosed with vitamin D deficiency						
No	Ref					
Yes	1.422	1.048–1.930	0.024	0.834	0.524–1.327	0.444
Vitamin D supplementation						
No	Ref					
Yes	1.480	1.090–2.010	0.012	1.038	0.683–1.579	0.861
Vitamin D supplementation during pregnancy						
No	Ref					
Yes	1.205	0.880–1.650	0.245			
Supplement infant with vitamin D						
No	Ref					
Yes	0.653	0.470–0.906	0.011	0.695	0.458–1.055	0.087
Supplement children without needed with vitamin D						
No	Ref					
Yes	1.420	1.048–1.924	0.024	1.414	1.007–1.987	0.046

## Data Availability

The data presented in this study are available on request from the corresponding author.

## Supplementary Materials

The following supporting information can be downloaded at: https://www.mdpi.com/article/10.3390/diseases13020058/s1. File S1: Questionnaire Title.

## References

[1] Holick M.F. (2011). Vitamin D: Evolutionary, Physiological and Health Perspectives. Curr. Drug Targets.

[2] Voulgaridou G., Papadopoulou S.K., Detopoulou P., Tsoumana D., Giaginis C., Kondyli F.S., Lymperaki E., Pritsa A. (2023). Vitamin D and Calcium in Osteoporosis, and the Role of Bone Turnover Markers: A Narrative Review of Recent Data from RCTs. Diseases.

[3] Uday S., Högler W. (2017). Nutritional Rickets and Osteomalacia in the Twenty-First Century: Revised Concepts, Public Health, and Prevention Strategies. Curr. Osteoporos. Rep..

[4] Hou Y.-C., Wu C.-C., Liao M.-T., Shyu J.-F., Hung C.-F., Yen T.-H., Lu C.-L., Lu K.-C. (2018). Role of Nutritional Vitamin D in Osteoporosis Treatment. Clin. Chim. Acta.

[5] Ismailova A., White J.H. (2022). Vitamin D, Infections and Immunity. Rev. Endocr. Metab. Disord..

[6] Chanet A., Verlaan S., Salles J., Giraudet C., Patrac V., Pidou V., Pouyet C., Hafnaoui N., Blot A., Cano N. (2017). Supplementing Breakfast with a Vitamin D and Leucine-Enriched Whey Protein Medical Nutrition Drink Enhances Postprandial Muscle Protein Synthesis and Muscle Mass in Healthy Older Men. J. Nutr..

[7] Detopoulou P., Papadopoulou S.K., Voulgaridou G., Dedes V., Tsoumana D., Gioxari A., Gerostergios G., Detopoulou M., Panoutsopoulos G.I. (2022). Ketogenic Diet and Vitamin D Metabolism: A Review of Evidence. Metabolites.

[8] Umar M., Sastry K.S., Chouchane A.I. (2018). Role of Vitamin D Beyond the Skeletal Function: A Review of the Molecular and Clinical Studies. Int. J. Mol. Sci..

[9] Cui A., Zhang T., Xiao P., Fan Z., Wang H., Zhuang Y. (2023). Global and Regional Prevalence of Vitamin D Deficiency in Population-Based Studies from 2000 to 2022: A Pooled Analysis of 7.9 Million Participants. Front. Nutr..

[10] Al-Qahtani S.M., Shati A.A., Alqahtani Y.A., Dawood S.A., Siddiqui A.F., Zaki M.S.A., Khalil S.N. (2022). Prevalence and Correlates of Vitamin D Deficiency in Children Aged Less than Two Years: A Cross-Sectional Study from Aseer Region, Southwestern Saudi Arabia. Healthcare.

[11] Voortman T., van den Hooven E.H., Heijboer A.C., Hofman A., Jaddoe V.W., Franco O.H. (2015). Vitamin D Deficiency in School-Age Children Is Associated with Sociodemographic and Lifestyle Factors1, 2, 3. J. Nutr..

[12] Kyriakaki A., Fragkoulis E. (2019). The Vitamin D Paradox: High Prevalence of Deficiency in Sunny Athens (Greece). Ann. Res. Hosp..

[13] Lips P., Cashman K.D., Lamberg-Allardt C., Bischoff-Ferrari H.A., Obermayer-Pietsch B., Bianchi M.L., Stepan J., Fuleihan G.E.-H., Bouillon R. (2019). Current Vitamin D Status in European and Middle East Countries and Strategies to Prevent Vitamin D Deficiency: A Position Statement of the European Calcified Tissue Society. Eur. J. Endocrinol..

[14] Cashman K.D. (2020). Vitamin D Deficiency: Defining, Prevalence, Causes, and Strategies of Addressing. Calcif. Tissue Int..

[15] Alagöl F., Shihadeh Y., Boztepe H., Tanakol R., Yarman S., Azizlerli H., Sandalci O. (2000). Sunlight Exposure and Vitamin D Deficiency in Turkish Women. J. Endocrinol. Investig..

[16] Cashman K.D. (2022). 100 years of vitamin D: Global differences in vitamin D status and dietary intake: A review of the data. Endocr. Connect..

[17] Mithal A., Wahl D.A., Bonjour J.-P., Burckhardt P., Dawson-Hughes B., Eisman J.A., El-Hajj Fuleihan G., Josse R.G., Lips P., Morales-Torres J. (2009). Global Vitamin D Status and Determinants of Hypovitaminosis D. Osteoporos. Int..

[18] Haq A., Wimalawansa S.J., Pludowski P., Anouti F.A. (2018). Clinical Practice Guidelines for Vitamin D in the United Arab Emirates. J. Steroid Biochem. Mol. Biol..

[19] Alibrahim H., Swed S., Bohsas H., Abouainain Y., Jawish N., Diab R., Ishak A., Saleh H.H., Nasif M.N., Arafah R. (2024). Assessment the Awareness of Vitamin D Deficiency among the General Population in Syria: An Online Cross-Sectional Study. BMC Public Health.

[20] Janda M., Youl P., Bolz K., Niland C., Kimlin M. (2010). Knowledge about Health Benefits of Vitamin D in Queensland Australia. Prev. Med..

[21] Zadka K., Pałkowska-Goździk E., Rosołowska-Huszcz D. (2018). The State of Knowledge about Nutrition Sources of Vitamin D, Its Role in the Human Body, and Necessity of Supplementation among Parents in Central Poland. Int. J. Environ. Res. Public Health.

[22] De Vriendt T., Matthys C., Verbeke W., Pynaert I., De Henauw S. (2009). Determinants of Nutrition Knowledge in Young and Middle-Aged Belgian Women and the Association with Their Dietary Behaviour. Appetite.

[23] O’Connor C., Glatt D., White L., Revuelta Iniesta R. (2018). Knowledge, Attitudes and Perceptions towards Vitamin D in a UK Adult Population: A Cross-Sectional Study. Int. J. Environ. Res. Public Health.

[24] EU Register of Health Claims—European Commission. https://food.ec.europa.eu/food-safety/labelling-and-nutrition/nutrition-and-health-claims/eu-register-health-claims_en.

[25] Office of Dietary Supplements—Vitamin D. https://ods.od.nih.gov/factsheets/VitaminD-HealthProfessional/.

[26] Bujang M.A., Sa’at N., Sidik T.M.I.T.A.B., Joo L.C. (2018). Sample Size Guidelines for Logistic Regression from Observational Studies with Large Population: Emphasis on the Accuracy Between Statistics and Parameters Based on Real Life Clinical Data. Malays. J. Med. Sci..

[27] Saleh A., Alhadhrami J.S., Al Ramahi M.S., Albloushi H.A., Hijazi R., Abboud M., Papandreou D. (2020). Emirati Adults Have a Higher Overall Knowledge on Vitamin D Compared to Tourists. Front. Psychol..

[28] Kambal N., Abdelwahab S., Albasheer O., Taha S., Abdelrahman N., Bani I., Alsayegh A., Shammaky E., Duwayri N., Alhazmi A. (2023). Vitamin D Knowledge, Awareness and Practices of Female Students in the Southwest of Saudi Arabia: A Cross-Sectional Study. Medicine.

[29] Alemu E., Varnam R. (2012). Awareness of Vitamin D Deficiency among At-Risk Patients. BMC Res. Notes.

[30] Tanna N.K., Karki M., Webber I., Alaa A., El-Costa A., Blair M. (2023). Knowledge, Attitudes, and Practices Associated with Vitamin D Supplementation: A Cross-Sectional Online Community Survey of Adults in the UK. PLoS ONE.

[31] Boland S., Irwin J.D., Johnson A.M. (2015). A Survey of University Students’ Vitamin D-Related Knowledge. J. Nutr. Educ. Behav..

[32] Kung A.W.C., Lee K.-K. (2006). Knowledge of Vitamin D and Perceptions and Attitudes toward Sunlight among Chinese Middle-Aged and Elderly Women: A Population Survey in Hong Kong. BMC Public Health.

[33] Ho-Pham L., Nguyen M. (2012). Survey on Knowledge and Attitudes on Vitamin D and Sunlight Exposure in an Urban Population in Vietnam. J. ASEAN Fed. Endocr. Soc..

[34] Vu L.H., van der Pols J.C., Whiteman D.C., Kimlin M.G., Neale R.E. (2010). Knowledge and Attitudes about Vitamin D and Impact on Sun Protection Practices among Urban Office Workers in Brisbane, Australia. Cancer Epidemiol. Biomark. Prev..

[35] Zhou M., Zhuang W., Yuan Y., Li Z., Cai Y. (2016). Investigation on Vitamin D Knowledge, Attitude and Practice of University Students in Nanjing, China. Public Health Nutr..

[36] Alfadly S., Anaam M., Alsahali S., Alshammari M., Almunef M., Almogbel Y., Alramadi I., Alodilah A. (2024). Knowledge, Attitude, and Practice (KAP) towards Vitamin D Deficiency among Adult Population in Qassim, Saudi Arabia. Open Public Health J..

[37] Aljefree N., Lee P., Ahmed F. (2017). Exploring Knowledge and Attitudes about Vitamin D among Adults in Saudi Arabia: A Qualitative Study. Healthcare.

[38] Uzrail A.H., Assab M.A., Alkalbani R., Kofahi R.A., Kadhim A. (2021). Knowledge, Attitude and Practice (KAP) Towards Vitamin D Deficiency in the Jordanian Adult Population: A Cross-Sectional Study. Res. J. Med. Sci..

[39] Arora H., Dixit V., Srivastava N. (2016). Evaluation of knowledge, practices of vitamin D and attitude towards sunlight among Indian students. Asian J. Pharm. Clin. Res..

[40] Malek O., Hajjeh M., Safiah M.H., Alourfi Z. Research Square—Assessing Vitamin D Knowledge Among Syrian Adults: A Population-Based Cross-Sectional Study. 2023. https://www.researchsquare.com/article/rs-2970287/v2.

[41] Babelghaith S., Ali W., Al-Zaaqi M.A., Al-Malki A.S., Al-Amri F.D., Alfadly S., Alghadeer S., Alarifi M.N. (2017). Knowledge and Practice of Vitamin d Deficiency among People Lives in Riyadh, Saudi Arabia—A Cross-Sectional Study. Biomed. Res..

[42] Alamoudi L.H., Almuteeri R.Z., Al-Otaibi M.E., Alshaer D.A., Fatani S.K., Alghamdi M.M., Safdar O.Y. (2019). Awareness of Vitamin D Deficiency among the General Population in Jeddah, Saudi Arabia. J. Nutr. Metab..

[43] Kanis J.A., Cooper C., Rizzoli R., Reginster J.-Y. (2019). European Guidance for the Diagnosis and Management of Osteoporosis in Postmenopausal Women. Osteoporos. Int..

[44] Saadeh R., Jumaa D., Elsalem L., Batieha A., Jaddou H., Khader Y., El-Khateeb M., Ajlouni K., Allouh M.Z. (2022). Osteoporosis among Postmenopausal Women in Jordan: A National Cross-Sectional Study. Int. J. Environ. Res. Public Health.

[45] Eid Al Agha A., Alorabi S.H. (2016). Awareness of Vitamin D and Its Deficiency in Jeddah Population, Saudi Arabia. J. Comm. Pub Health Nurs..

[46] Al-Ghraibawi S.J., Al-Ghabban S.I., Al-Zubaidy R.D. (2019). Knowledge and practices regarding vitamin d deficiency among women attending imam hussein medical city in karbala 2018. Int. J. Curr. Pharm. Res..

[47] Tariq A., Khan S.R., Basharat A. (2020). Assessment of Knowledge, Attitudes and Practice towards Vitamin D among University Students in Pakistan. BMC Public Health.

