# Supplementary material for: Knowledge and Predictors of Vitamin D Awareness Among Greek Women: A Cross-Sectional Study
Source: Diseases. 2025 Feb 15;13(2):58. doi: 10.3390/diseases13020058 (PMC11854788; doi:10.3390/diseases13020058)
Supplement: Supplementary file 1 [file diseases-13-00058-s001.zip › diseases-3452349-supplementary.pdf]

1. **Write your age in years (e.g., 35)** 1
2. **Write your weight in kilograms (e.g., 65)** 2
3. **Write your height in centimeters (e.g., 170)** 3
4. **What is the population of the area where you live?** 4
   - Less than 2,000 inhabitants 5
   - 2,000-10,000 inhabitants 6
   - 10,000-50,000 inhabitants 7
   - More than 50,000 inhabitants 8
5. **How many children under the age of 18 do you have?** 9
   - 1 10
   - 2 11
   - >3 12
   - None 13
6. **Write the age of your oldest child (e.g., 16):** 14
7. **What is your level of education?** 15
   - Primary education (Elementary) 16
   - Secondary education (High School, Lyceum) 17
   - Higher education (University, Polytechnic) 18
8. **What is your occupation?** (e.g., Household, etc.) 19
9. **What is the net monthly income of your household?** 20
   - Up to 1,000 euros 21
   - 1,000-2,000 euros 22
   - 2,000-3,000 euros 23
   - More than 3,000 euros 24
10. **Do you know the functions of Vitamin D that make it essential for the human body?** 25
11. **Do you know some diseases that are associated with or treated by Vitamin D?** 26
12. **In which age group of children are the greatest need for Vitamin D?** 27
    - 0-6 years 28
    - 7-12 years 29
    - 13-18 years 30
    - I don't know 31
13. **Select the food or food group that you know contains Vitamin D.** 32
    - Dairy products 33
    - Cereals/Bakery products 34
    - Fruits 35
    - Vegetables 36
    - Eggs 37
    - Meat 38
    - Poultry 39

Fish

Seafood (e.g., sardines, squid, shrimp, etc.)

Nuts

I don’t know / I’m not sure

14. **How often do you consume these foods at the family table?**

Dairy products (e.g., milk, cheese, yogurt)

Bakery products

Fruits

Vegetables

Eggs

Meat

Poultry

Fish

Seafood (e.g., sardines, squid, shrimp, etc.)

Nuts

15. **How many times have you been tested for Vitamin D levels in your body?**

Once

Twice

More than twice

Never

I don't know

16. Have you been diagnosed with vitamin D deficiency or insufficiency in your body at least once in your lifetime?

Yes

No

I haven't been tested

17. **Have you been taking a vitamin D supplement for at least one period outside of pregnancy?**

Yes

No

18. **Did you take a supplement for vitamin D during a pregnancy?**

Yes

No

19. **Have you ever given Vitamin D supplements to your children without need?**

Yes

No

20. Have you ever given Vitamin D supplements to your children without need?

Yes

No
